# Hypoxia Regulates mTORC1-Mediated Keratinocyte Motility and Migration via the AMPK Pathway

**DOI:** 10.1371/journal.pone.0169155

**Published:** 2017-01-09

**Authors:** Tiantian Yan, Junhui Zhang, Di Tang, Xingyue Zhang, Xupin Jiang, Liping Zhao, Qiong Zhang, Dongxia Zhang, Yuesheng Huang

**Affiliations:** Institute of Burn Research, State Key Laboratory of Trauma, Burns and Combined Injury, Southwest Hospital, Third Military Medical University, Chongqing, China; Suzhou University, CHINA

## Abstract

Keratinocyte migration, the initial event and rate-limiting step in wound healing, plays a vital role in restoration of the intact skin barrier, also known as re-epithelialization. After acute tissue injury, hypoxic microenvironment gradually develops and acts as an early stimulus to initiate the healing process. Although we have previously found that hypoxia induces keratinocyte migration, the underlying mechanism remains unknown. Here, we first observed that hypoxia increased mTORC1 activity. Recombinant lentivirus vector and Rapamycin were used for silencing mTORC1 in HaCaT cells and primary mouse keratinocytes (MKs). Using cell migration assay and a Zeiss chamber equipped with imaging system, we also demonstrated that mTORC1 downregulation reversed hypoxia-induced keratinocyte motility and lateral migration. Importantly, hypoxia-activated mTORC1 was accompanied by the AMPK downregulation, and we found that the AMPK pathway activators Metformin (Met) and 5-Aminoimidazole-4-carboxamide 1-β-D-ribofuranoside (AICAR) decreased the mTORC1 activity, cell motility and lateral migration. Thus, our results suggest that hypoxia regulates mTORC1-mediated keratinocyte motility and migration via the AMPK pathway.

## Introduction

Wound healing is a dynamic and well-ordered biological process that requires the spatial and temporal orchestration of several distinct components, including coagulation, inflammation, re-epithelialization, contraction and remodeling [1]. The essential feature of successful wound healing is the reestablishment of the intact epidermal barrier. Thus, wound epithelialization, also called re-epithelialization, is the key and defining feature of wound repair. The term “re-epithelialization” refers to an intricate process that the keratinocytes migrate from wound margins to resurface the wounded area. During this process, keratinocyte migration into the wound is the initial event and rate-limiting step [2], since defects in migration, but not in differentiation or proliferation, are closely related with the non-healing wounds.

Under normal conditions, keratinocytes develop a mechanical barrier against chemical stimulus and microorganism through terminal differentiation. During wound healing, a complex balance of genes and signals are regulated in a temporal and spatial manner to promote keratinocyte motility and complete re-epithelialization, including desmosomes, cytokines, integrins, and oxygen tension, among other components. As an essential necessity for cells, oxygen has a central role in oxidative phosphorylation, enzymatic reactions, and signal pathways. Acute skin injury caused vascular disruption and microcirculation interruption that leads to low oxygen tension (hypoxia). The hypoxic microenvironment is exacerbated by high oxygen consumption of the active cells in granulation tissue [3, 4]. The responses of cells to hypoxia have received considerable attention. These include modulation of angiogenesis, cell proliferation, transcription, translation, and vascular remodeling. Coincidentally, hypoxia found in the healing margin of normal wounds was related to cell migration, but not to proliferation or inflammatory infiltration. Therefore, hypoxia may be an early stimulus for the initiation of cell motility and migration through unclear mechanisms.

Mammalian target of rapamycin (mTOR) is an evolutionarily conserved kinase that exists in two complexes: mTORC1 and mTORC2. The vital role of mTOR in integrating information from extracellular nutrients, energy and growth factors in the coordination of cellular physiology and molecular biology has been well documented [5, 6]. Mounting evidence shows that mTORC1 is crucial in the invasion and migration of multiple cancer cells [7, 8], but its role in keratinocytes remains to be elucidated. mTORC1 (pS6) is up-regulated in the epithelial tongue, corresponding to the migratory areas of normal wounds [9]. Considering that the appearance of hypoxia is also strongly correlated with the mTORC1 (pS6) up-regulation and keratinocyte migration [10] during wound repair, we reasoned that mTORC1 up-regulation may be related to hypoxic keratinocyte migration through unknown mechanisms.

Responses to mild hypoxia at the molecular level are hallmarks of adaptation and survival under oxygen-deficient conditions. Adenosine 5‘-monophosphate (AMP)-activated protein kinase (AMPK), which has an important role under hypoxia, plays different roles in a context- and tissue-dependent manner [11–13]. In addition to its role in metabolism, AMPK has been strongly linked to cell migration, playing variable roles in different cells and contexts [14, 15]. Recently, Met, an AMPK activator, has been shown to reduce cell proliferation and delay wound healing [16], indicating that AMPK may be involved in cellular biology in keratinocyte during the process of wound repair. Additionally, a growing number of studies have identified an interactional relationship between AMPK and mTORC1 [17, 18]. These reports and observed results raised the hypothesis that AMPK and mTORC1 may be both involved in the possible relationship between hypoxia and keratinocyte migration.

In the present study, we used primary mouse keratinocytes (MKs) and HaCaT cells to identify the roles of hypoxia in the regulation of mTORC1 activity and keratinocyte migration. We observed that mTORC1 activity was up-regulated in hypoxic keratinocytes and that its suppression was sufficient to reverse hypoxia-induced keratinocyte motility and lateral migration. The AMPK signaling pathway suppressed mTORC1 activity, cell motility and lateral migration. Collectively, our results have suggested that hypoxia-activated mTORC1 participates in the regulation of keratinocyte migration and that AMPK suppression contributes to mTORC1 activation. Our findings reveal the distinct effect of AMPK and the mTORC1 signaling pathway in hypoxic keratinocytes and their critical roles in regulating cell motility and migration. These results provide novel insights into mTORC1 regulation and its effect on keratinocyte migration during wound healing.

## Materials and Methods

### Ethics statement

All of the animal-based investigations herein were approved by the Animal Experiment Ethics Committee of the Third Military Medical University and were performed in accordance with the Guide for the Care and Use of Laboratory Animals published by the National Institutes of Health (NIH Pub. No. 85–23, revised in 1996).

### Cell culture and hypoxia treatment

HaCaT cells were obtained from the Cell Bank of the Chinese Academy of Sciences (Beijing, China). Cells were cultured in RPMI 1640 medium (Hyclone, USA) supplemented with 100 U/ml penicillin, 100 mg/ml streptomycin, and 10% fetal bovine serum (Gibco, USA) in a 37°C humidified incubator at 5% CO_2_. New-born BALB/c mice were commercially purchased from the Laboratory Animal Center of the Third Military Medical University and sacrificed by cervical dislocation. Primary MKs were isolated from the skin of new-born BALB/c mice (postnatal day 1–3) as previously described [19]. Briefly, keratinocytes were obtained from the skin by incubation with a 0.25% trypsin/0.04% EDTA solution (Invitrogen, USA) at 4°C overnight and were then plated into collagen type IV-coated dishes. The cells were then maintained in keratinocyte serum-free medium (K-SFM medium) (Gibco, USA) supplemented with antibiotics in a humidified atmosphere containing 5% CO_2_ at 37°C.

A Forma Series II Water Jacket CO_2_ incubator (model: 3131; Thermo Scientific), which maintains a desired and precise culturing environment, kept CO_2_ at 5% and O_2_ at 2% by nitrogen displacement. Rapamycin (100 mM) and corresponding amounts of DMSO, used as an mTORC1 inhibitor and control, were added to the cultures, which were pre-incubated at 37°C for 4 h. Cells were incubated in the presence of the AMPK activators Met (500 μM) or AICAR (0.5 mM) throughout the experiment, unless otherwise indicated.

### Lentiviral transduction

The lentivirus for silencing Raptor (shRaptor) and non-targeting control (shNC) sequence were purchased from Shanghai GeneChem, Co. Ltd (Shanghai, China), and transfections were performed according to the manufacturer’s instructions.

### Western blotting

Cells were washed with ice-cold phosphate-buffered saline (PBS) and harvested on ice. The protein concentrations of the lysates were determined using an RCDC protein assay kit (Sigma, USA). A prestained standard protein molecular weight marker and the samples were loaded into the wells of 8% SDS–PAGE gels and transferred by electroblotting to polyvinylidene difluoride (PVDF) membranes. After blocking the membranes with 5% non-fat dried milk or bovine serum albumin (BSA), the desired bands were incubated overnight at 4°C with the corresponding primary antibodies. Horseradish peroxidase-conjugated secondary IgG (1:5,000, Proteintech, USA) was subsequently used as a secondary antibody. The results were analyzed using a ChemiDoc imaging system (Bio-Rad, USA). The primary antibodies used in this study were as follows: phospho-p70S6K (Thr389), p70S6K, phospho-4E-BP1 (Thr70), 4E-BP1, phospho-AMPKα (Thr172), AMPK, phospho-ACC (Ser79), ACC (1:1,000, Cell Signaling, USA).

### Cell motility assay and quantitative analysis

Cells were seeded into 24-well plates at a density of 0.5×10^4^/cm^2^. After appropriate cell attachment, cells with or without regulated mTORC1 or AMPK activity were transferred to a Zeiss CO_2_- and temperature-controlled chamber equipped with a Zeiss imaging system (Carl Zeiss Meditec, Jena, Germany), which performed time-lapse imaging. This system monitored single keratinocyte motility visually independent of cell proliferation and death. Images were acquired every 3 min for 3 h and were analyzed using NIH Image J software (http://rsb.info.nih.gov/ij/). The cell trajectories were imaged based on the positions of the cell nuclei at frame intervals of 3 min, and their trajectory speed (μm/min) was calculated as the total length (μm) of the trajectories divided by the time (minute), reflecting cell motility.

### Wound migration assay

A wound migration model of cultured cells was used as described in previous studies, with some modifications. Briefly, Culture-Inserts (Ibidi) were used to measure cell migration. Cell suspension at a density of 7×10^4^/mL (70 μL volume) was applied to each well of the Culture-Inserts. After the appropriate duration for cell attachment (24 h), the cells were incubated at 37°C for additional 2 h in the presence of mitomycin-C (5 μg/mL) [20] to inhibit cell proliferation. A cell-free gap of 500μm was created by removal of the Culture-Insert. Images were captured every 6 h using an inverted phase-contrast microscope. The percent of wound closure in five randomly chosen fields was calculated with NIH ImageJ software.

### Statistical analysis

The data are presented as the mean ± standard deviation (SD). SPSS 13.0 was used for statistical analysis, and statistical significance among multiple groups was evaluated by one-way ANOVA. P values < 0.05 were considered statistically significant.

## Results

### Hypoxia increases the activities of distinct mTORC1 downstream targets and their expression in the nucleus

**Fig 1:**
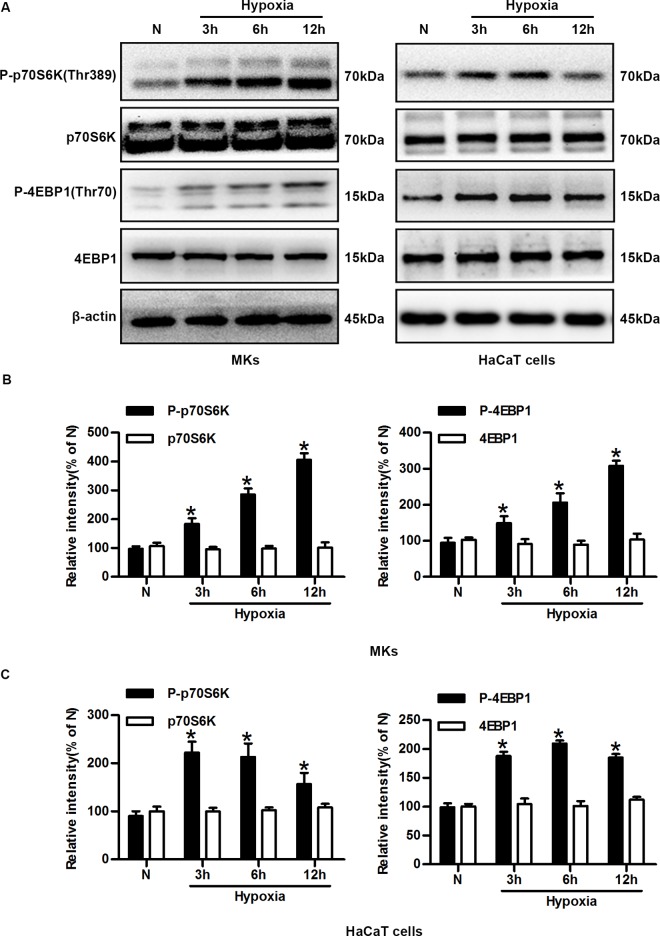
Hypoxia increases the activity of distinct mTORC1 downstream targets in keratinocytes. (A) Western blot showing total cell lysates of MKs and HaCaT cells exposed to hypoxia and probed for phosphorylated p70S6K (Thr389), p70S6K, phosphorylated 4E-BP1 (Thr70), 4E-BP1, and β-actin (loading control). (B) Quantitative analysis of expression of the aforementioned proteins in MKs. The graphs represent the mean ± SD (n = 3) of the relative integrated signals. N, normoxia. *P< 0.05 versus the N group. (C) Quantitative analysis of the expression of these proteins in HaCaT cells. The graphs represent the mean ± SD (n = 3) of the relative integrated signals. N, normoxia. *P< 0.05 versus the N group.

The activity of mTORC1 can be measured by analyzing phosphorylation of the direct downstream target of ribosomal protein kinase S6 (p70S6K) at Thr389 and eukaryotic initiation factor 4E binding protein 1 (4E-BP1) at Thr70. Thus, we performed time-course experiments in which MKs and HaCaT cells were exposed to hypoxia (2% O_2_) for different durations. The phosphorylation of p70S6K-Thr389 and 4E-BP1-Thr70 were then analyzed. As shown in Fig 1A, the phosphorylation of p70S6K (Thr389) and 4E-BP1 (Thr70) in MKs and HaCaT cells rapidly increased after exposure to hypoxic conditions and remained elevated compared with normoxic cells. The total p70S6K and 4E-BP1 levels remained unchanged under hypoxia. The average quantifications from cumulative experiments shown in Fig 1B and 1C indicated that the p70S6K and 4E-BP1 activities were significantly increased.

Subcellular localization, constituting the environment in which proteins act, is a crucial determinant of protein function and regulation. We examined whether hypoxia (2% O_2_) influences the cellular localization of mTORC1 downstream substrates. Under all tested conditions, phospho-p70S6K was localized to only distinct nuclear structures, whereas phospho-4E-BP1 was detected predominantly in nuclei, as shown by immunostaining in S1 Fig. Exposure to hypoxic conditions for 6 h clearly increased the phospho-p70S6K and phospho-4E-BP1 expression levels in the nuclei. Thus, we found that hypoxia rapidly promoted the phosphorylation of p70S6K and 4E-BP1 and increased their expression levels in nuclei. These results indicated that mTORC1 is activated by hypoxia.

### The mTORC1 pathway is required for hypoxia-induced keratinocyte motility and migration

A tremendous amount of data has established how mTORC1 controls different processes involved in disease, such as the migration and invasion of multiple types of tumour cells. Therefore, we hypothesized that mTORC1 might regulate single-keratinocyte motility and lateral migration under hypoxia.

**Fig 2:**
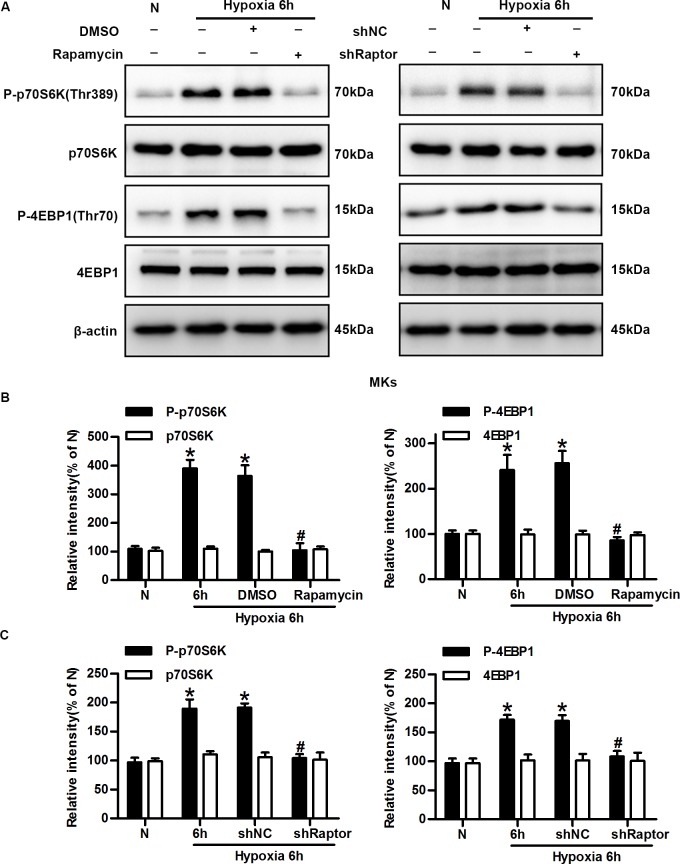
mTORC1 is inhibited by rapamycin and shRNA against Raptor. (A) Keratinocytes were incubated under normoxic and hypoxic conditions in the presence or absence of rapamycin (100 mM, SB) or the shRNA against Raptor (shRaptor). Western blotting was performed to analyze the expression of phosphorylated p70S6K (Thr389), p70S6K, phosphorylated 4E-BP1 (Thr70), 4E-BP1, and β-actin (loading control). (B) The graphs represent the mean ± SD (n = 3) of the relative integrated signals. N, normoxia. *P< 0.05 versus the N group. ^#^P< 0.05 versus the hypoxia 6 h + DMSO group. (C) The graphs represent the mean ± SD (n = 3) of the relative integrated signals. N, normoxia. *P< 0.05 versus the N group. ^#^P< 0.05 versus the hypoxia 6 h + shNC group.

To determine the role of mTORC1 in keratinocyte migration, we first used rapamycin, a classical inhibitor of mTORC1, to block its activity. As previously shown, exposure to hypoxic conditions for 6 h resulted in markedly increased phosphorylation of p70S6K and 4E-BP1, which was inhibited by rapamycin (Fig 2A and 2B). Next, Raptor, an essential component of mTORC1, was selectively silenced. Keratinocytes with stable shRNA-mediated knockdown of Raptor was generated and exhibited > 90% reduction in expression of the targeted protein (S2 Fig). Cells expressing shRaptor had significantly reduced levels of phospho-p70S6K and phospho-4E-BP1 compared with hypoxic cells (with or without shNC), whereas total p70S6K and 4E-BP1 levels were not affected by hypoxia or shRNA (Fig 2A and 2C).

**Fig 3:**
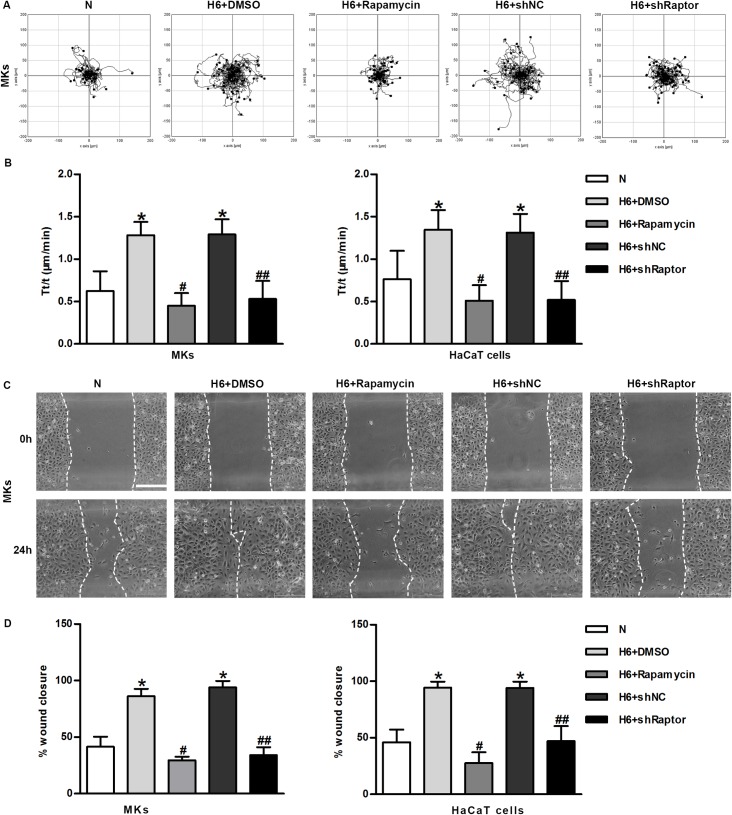
The mTORC1 pathway is required for hypoxia-induced keratinocyte motility and migration. (A) The movement trajectories of cells with mTORC1 inhibition under normoxic or hypoxic culture condition. (B) Statistical analysis of the trajectory speeds of mTORC1-inhibited MKs and HaCaT keratinocytes. The data were from at least 100 cells in 3 independent experiments and are shown as the mean ± SD. N, normoxia. *P< 0.05 versus the N group. ^#^P< 0.05 versus the hypoxia 6 h + DMSO group. ^##^P< 0.05 versus the hypoxia 6 h + shNC group. (C) Wound-healing assays were performed using Culture-Inserts. The cell migration of normoxic and hypoxic MKs with mTORC1 inhibition were recorded for 24 h after wounding. Scalebar = 200 μm. The wound closure (%) was the reduction in the original cell-free wound area. (D) The graph represents the mean ± SD (n = 3). N, normoxia. *P< 0.05 versus the N group. ^#^P< 0.05 versus the hypoxia 6 h + DMSO group. ^##^P< 0.05 versus the hypoxia 6 h + shNC group.

Migration is a critical step in the initial process of wound repair. A Zeiss imaging system was used to study the role of mTORC1 on single-keratinocyte motility independent of cell proliferation and survival. Wound migration assay was performed to observe keratinocytes lateral migration. Our results showed that the movement range of MKs exposed to hypoxia was greatly increased, thus indicating better motility. Both allosteric inhibition by rapamycin and the knockdown of Raptor markedly attenuated the hypoxia-induced cell motility (Fig 3A and 3B, S1 Movie). Similar results were also found in HaCaT cells (Fig 3B). The positive role of mTORC1 in hypoxia-induced lateral migration was further confirmed in an *in vitro* wound migration assay. After 24 h, hypoxic keratinocytes (H + DMSO, H + shNC) migrated into nearly 100% of the original non-cell-covered areas, whereas mTORC1-inhibited keratinocytes (H + rapamycin, H + shRaptor) migrated into only 25% of the original cell-free areas under hypoxic conditions (Fig 3C and 3D). Similar results were observed in treated HaCaT cells (Fig 3D). In summary, the pharmacological blockade of mTOR signaling using rapamycin and the genetic modification of an essential component of mTORC1 significantly inhibited single-keratinocyte motility and lateral migration. The above data have revealed that mTORC1 activation is essential for hypoxia-enhanced keratinocyte motility and migration, although the underlying molecular mechanism remains to be elucidated.

### Hypoxia mediates time-dependent, sustained suppression of the AMPK signaling pathway in keratinocytes

**Fig 4:**
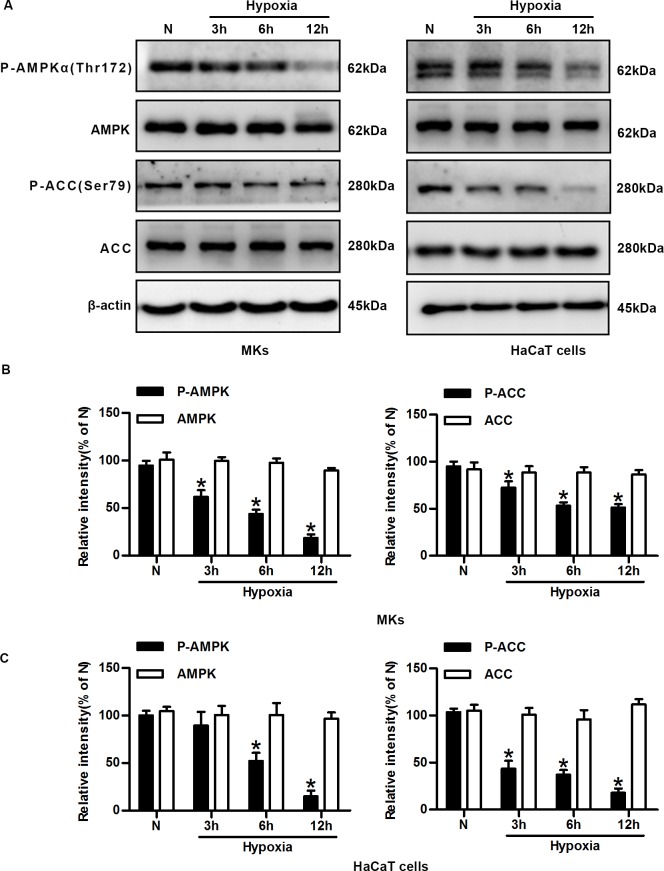
Hypoxia mediates time-dependent, sustained suppression of the AMPK signalling pathway in keratinocytes. (A) Representative cropped blots of phosphorylated AMPKα (Thr172), AMPK, phosphorylated ACC (Ser79), ACC, and β-actin (loading control) in MKs and HaCaT cells under normoxic and hypoxic conditions. (B) The graphs represent the mean ± SD of the relative integrated signals in MKs. N, normoxia. *P< 0.05 versus the N group. (C) The statistical graphs represent the mean ± SD of the relative integrated signals in HaCaT cells. N, normoxia. *P< 0.05 versus the N group.

Our finding demonstrated that mTORC1 activation plays crucial roles in the regulation of single-cell motility and lateral migration during hypoxia, we then study the mechanism of mTORC1 activation in hypoxic keratinocytes. The phosphorylation statuses of AMPKα and acetyl-CoA carboxylase (ACC) were examined by immunoblotting analysis using commercially available antibodies to assess AMPK activity. Exposure of keratinocytes to hypoxia resulted in the dephosphorylation (inactivation) of AMPKα and ACC in a time-dependent manner (Fig 4A). The quantification from cumulative experiments confirmed that the expression levels of phosphor-AMPKα and phosphor-ACC inversely correlated with the duration of hypoxic treatment in MKs and HaCaT cells (Fig 4B and 4C). The total AMPK and ACC levels remained unchanged under hypoxia. Considering the early reports demonstrating that AMPK can suppress mTORC1, we reasoned that there might be a causal relationship between AMPK and mTORC1.

### AMPK activation reverses hypoxia-induced mTORC1 activation

**Fig 5:**
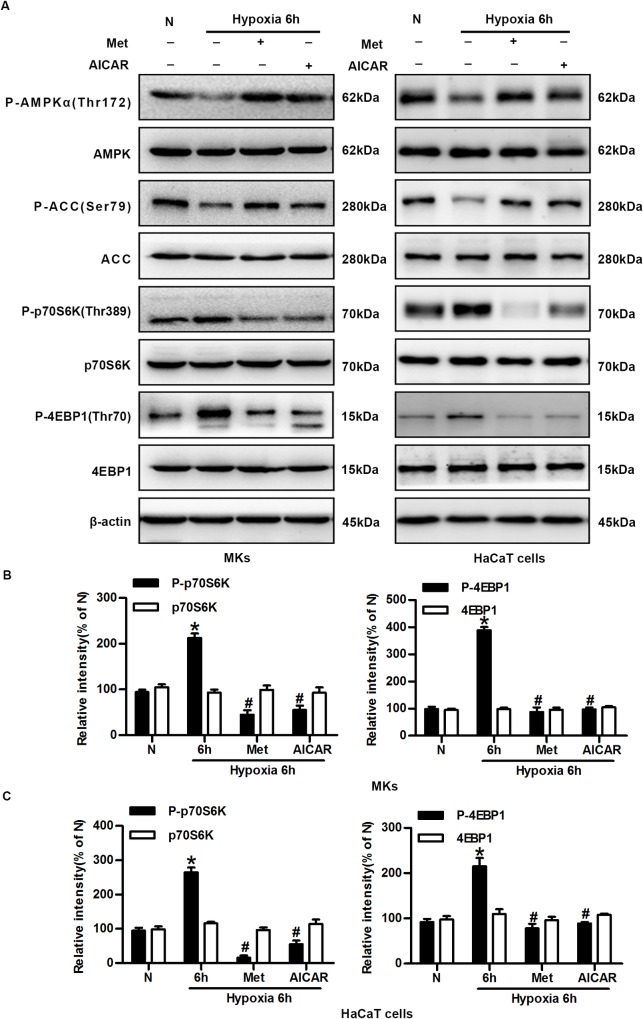
AMPK activation reverses hypoxia-induced mTORC1 activation. (A) Western blot analysis of the effects of AMPK pathway activators Met and AICAR on the expression of phosphorylated p70S6K (Thr389), p70S6K, phosphorylated 4E-BP1 (Thr70), 4E-BP1, phosphorylated AMPKα (Thr172), AMPK, phosphorylated ACC (Ser79), ACC and β-actin (loading control). (B) The graphs represent the mean ± SD (n = 3) of the relative integrated signals in MKs. N, normoxia. *P< 0.05 versus the N group. ^#^P< 0.05 versus the hypoxia 6 h group. (C) The statistical graphs represent the mean ± SD (n = 3) of the relative integrated signals in HaCaT cells. N, normoxia. *P< 0.05 versus the N group. ^#^P< 0.05 versus the hypoxia 6 h group.

To study the possible causal relationship between AMPK suppression and mTORC1 activation, keratinocytes were treated with the AMPK activators Met and AICAR. They activated AMPK effectively in MKs, as shown by the increased phosphorylation of AMPKα and ACC after exposure to hypoxic conditions for 6 h. Meanwhile, the expression levels of phosphorylated p70S6K and 4E-BP1 were consistently decreased (up to 90%) by Met and AICAR, indicating the suppression of mTORC1 activity. The total levels of AMPK, ACC, p70S6K, and 4E-BP1 were not affected by either hypoxia or the AMPK activators (Fig 5A and 5B). Met and AICAR had similar effects on mTORC1 in HaCaT cells (Fig 5A and 5C). These results suggest that the AMPK pathway negatively regulates the mTORC1 activity in hypoxic keratinocytes.

### The AMPK suppression is involved in hypoxia-enhanced motility and migration

**Fig 6:**
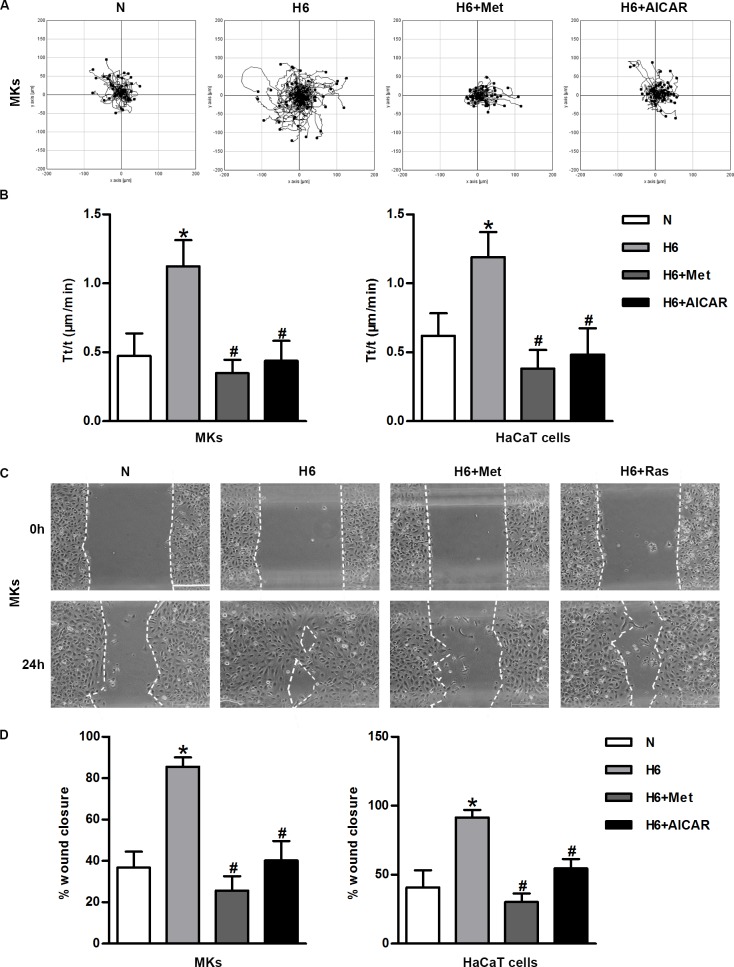
AMPK is involved in keratinocyte migration under hypoxia. (A) The representative migration trajectories of MKs under normoxic or hypoxic conditions in the presence or absence of Met and AICAR. (B) Statistical analysis of the trajectory speeds of AMPK-activated MKs and HaCaT cells. The data were from at least 100 cells in 3 independent experiments and were shown as the mean ± SD. N, normoxia. *P< 0.05 versus the N group. ^#^P< 0.05 versus the hypoxia 6 h group. (C) Cell migration was measured by using Culture-Inserts. The wound closure (%) was the reduction in the original non-covered wound area. Scalebar = 100 μm. (D) The panels represent the covered wound areas and show the mean ± SD (n = 3). N, normoxia. *P< 0.05 versus the N group. ^#^P< 0.05 versus the hypoxia 6 h group.

To further study the role of AMPK in the augmented motility and migration of hypoxic keratinocytes, we analyzed the movement of these cells using a Zeiss imaging system and wound migration assays with or without an AMPK pharmacological activator. Images recording cell trajectories showed that hypoxia greatly increased movement range of keratinocytes, whereas the pharmacological activator of AMPK significantly reduced cell motility (Fig 6A, S2 Movie). The statistical analysis of the velocity confirmed the inhibitory effect of AMPK activation on the keratinocyte motility, because the corresponding histograms for the MKs and HaCaT cells showed 2-fold reductions in their trajectory speeds in the presence of Met and AICAR (Fig 6B). The similar effect of AMPK activation on migratory capacity was observed using wound healing assays. Hypoxic keratinocytes covered nearly 90% of the original cell-free area, while the areas covered by cells treated with Met and AICAR were only 30% and 50%, respectively; the statistical analysis of the monolayer migration assays showed the similar result (P< 0.05) (Fig 6C and 6D). Taken together, these results demonstrate that the AMPK pathway is negatively correlated with hypoxia-induced keratinocyte migration and that this correlation contributes to the crucial role of mTORC1 in regulating keratinocyte migration after hypoxia.

## Discussion

Re-epithelialization, the ultimate goal and defining sign of a healed wound, includes three overlapping keratinocyte functions: migration, proliferation, and differentiation. Keratinocytes migration, the most limiting process, has received considerable attention. Hypoxic microenvironment is vital and significant occurrence to which keratinocytes are exposed. In this study, we investigated the mechanisms responsible for the augmented motility and migration of keratinocytes in the mild hypoxic microenvironment and found that mTORC1 activation, which was mediated by AMPK suppression, played a vital role in hypoxia-induced cell migration. First, we demonstrated that hypoxia-induced mTORC1 activation was greatly involved in keratinocytes motility and migration (Figs 1–3, S1 Fig, S1 Movie). Second, we found that the hypoxia suppressed the AMPK signaling pathway in a time-dependent manner (Fig 4). Third, we showed that AMPK activation via Met and AICAR reversed hypoxia-induced mTORC1 activation, keratinocytes motility, and migration (Figs 5 and 6, S2 Movie). Our results clarify the role of mTORC1 in hypoxic keratinocytes, as the findings of mTOR studies using different tissues or cells have showed differing results. Earlier studies found that hypoxia activated mTOR signaling [21] and resulted in HIF-1α stabilization [22], angiogenesis [23], and proliferation [24], meanwhile hypoxia appeared to inhibit mTOR signaling pathway, as demonstrated by inactivation of the S6K1 and 4E-BP1 [25].

The disruption of skin integrity with the loss of epithelial cells continuity results in the activation of an intricate process aiming to restore an intact epidermal barrier, and keratinocyte migration is a critical step in this process. Migrating cells at the epithelial tongue are likely to encounter mild hypoxia due to decreased oxygen supply and increased oxygen demands. After acute injury, hypoxic stress induces or suppresses certain genes and protein synthesis, which is the essential strategy for adaptation to hypoxic environment. Pimonidazole, a specific tissue hypoxia marker, is used to detect moderate hypoxia at the leading edge of the healing area of epidermis [26]. Unlike complete anoxia, exposure of cells to mild hypoxia (1–2%O_2_, PO_2_ = 7–14 mmHg) can result in various consequences for metabolism, intracellular signaling, and survival. For instance, 1–2% O_2_ does not cause cells to undergo growth arrest or apoptosis [27], whereas cells undergo apoptosis at oxygen concentrations of closer to 0% [28]. In addition, an oxygen concentration of 0.5% or lower will limit the respiratory rate and decrease the ATP levels [29, 30]. In contrast, mild hypoxia elicits a well-coordinated series of events that influence gene expression and the phenotype. The early application of semi-occlusive dressings after injury has been shown to promote re-epithelialization substantially when compared with wounds allowed to air-dry [31, 32]. In conclusion, the moderate hypoxia microenvironment has important effects on the processes that occur during wound healing.

mTORC1 is critical in cell molecular activities. In the last years, many studies have established how the mTORC1 integrates the signals from extracellular environment in the regulation of cellular physiology and molecular biology, for instance, the mTORC1 pathway is pivotal in cancer cell invasion. During wound healing, upregulation of mTORC1 downstream targets are found in the epithelial tongue correspondent to the migratory areas of normal wounds. Coincidentally, hypoxia found in the healing margin of normal wounds was related to cell migration, but not to proliferation or inflammatory infiltration. These results prompted us to study the possibility that the hypoxic microenvironment promotes mTORC1 activation in migrating keratinocytes. In this study, we have demonstrated that hypoxia induced phosphorylation of the mTORC1 substrates p70S6K (Thr389) and 4E-BP1 (Thr70), and their expression in nuclear structures markedly increased. The phosphorylation of aforementioned proteins was inhibited by rapamycin pre-treatment and Raptor silencing. In line with these phosphorylation studies, Raptor silencing and rapamycin, thus the inhibition of mTORC1 signaling, all specifically and significantly inhibited single-cell motility and the lateral migration of keratinocytes under hypoxic conditions. These findings indicate that mTORC1 activity is very important in hypoxia-induced migration of keratinocytes. The role of mTORC1 in hypoxic keratinocytes is similar to that observed in hypoxic carcinoma cells whose mTORC1 activation is also associated with carcinoma progression and invasion. In our study, pre-treatment with rapamycin for 4 h, rather than prolonged treatment, did not inhibit mTORC2 assembly, in agreement with previous studies showing that mTORC2 (the RICTOR/mTOR complex) is insensitive to short-term treatment with rapamycin [33].

mTORC1 is traditionally believed to regulate cell growth and survival. Although our results highlight the importance of mTORC1 in migrating keratinocytes under hypoxia, the mechanisms that mTORC1 regulate hypoxic keratinocytes migration remain unclear. During the process of re-epithelialization, a series of orchestrated events transform a mature, differentiated keratinocyte into a migrating keratinocyte. The change from a sedentary cell to a motile cell involves changes in the cell attachments, keratins, matrix metalloproteinase, and tight junctions. Our study is just a preliminary exploration of the role of mTORC1 in migrating keratinocytes, and this finding raises the question that how mTORC1 links the expression of transcription factors and changes in migratory ability. Future studies may further elucidate the roles of mTORC1 in migrating keratinocytes.

In our study, we found that hypoxia mediated time-dependent, sustained suppression of the AMPK signaling pathway in keratinocytes and that this suppression was negatively correlated with the mTORC1 activation in hypoxic cells (Fig 4). The suppression of AMPK was verified by the phosphorylation of Thr172 in its α subunit and ACC, the rate-limiting enzyme in fatty acid synthesis. Accumulating data indicate that the AMPK pathway is involved in well-orchestrated signaling events triggered by hypoxia, and it is believed to sense energy stress and to be activated during sudden periods of acute stress. The different situation in hypoxic keratinocytes and the reasons for this phenomenon are quite intriguing. This discrepancy may potentially be explained by complex regulation of the AMPK network and the specific conditions. There are at least three (but probably more) kinases that phosphorylate AMPK at Thr172, thereby affecting its activity. The AMPK holoenzyme is also activated by allosteric regulation of the β subunit, with or without the phosphorylation of Thr172 in the α subunit [34]. Therefore, the functions of AMPK should be carefully considered depending on the specific context. In our situation, other factors instead of AMP/ATP play a leading role in suppressing the AMPK pathway, which remains to be explored in our further studies. Additionally, previous studies have shown that AMPK is involved in regulating the migration of different types of cells [14, 35]. However, there is no evidence showing that AMPK signaling is involved in hypoxic keratinocyte migration. Our results demonstrated that suppression of the AMPK pathway accelerated keratinocyte migration under hypoxia, whereas its pharmacological activation led to decreased cell movement and migration. Thus, this study has revealed that mild hypoxic microenvironment induces keratinocyte migration through the suppression of AMPK during the early stage of wound repair. New studies will continue to uncover the elegant network of events that result in the balance between AMPK suppression related migration and the energy stress.

Activation of mTORC1 should be fine regulated in consideration of energy level in hypoxic cells, so that cells can survive and maintain physiological function. As a biomarker for mTOR activation, the phosphorylation of mTOR-Ser2448 has been suggested to be an important part of a feedback mechanism that regulates its activity [36]. The p70S6 kinase is considered to be the dominant kinase responsible for the phosphorylation of mTOR-Ser2448 in cells [37]. Interestingly, we found that moderately hypoxic condition (2% O_2_) promoted the sustained and rapamycin-sensitive phosphorylation of mTOR (Ser2448) in keratinocytes, whereas total mTOR expression was not affected by hypoxia. These findings are consistent with other cell responses to hypoxia, preventing or delaying the onset of more severe hypoxia [38]. However, the functional significance of phosphorylated mTOR at Ser2448 is still unknown. Additionally, it is still unclear whether this feedback mechanism is positive or not and to what extent it affects mTORC1 [37, 39]. Further studies are necessary to decipher the relationship between hypoxia-induced mTOR phosphorylation (Ser2448) and mTOR function.

In conclusion, our results have revealed that mTORC1 activation caused by the AMPK suppression is closely involved in mild hypoxia-induced single-cell motility and lateral migration. These findings provide a new perspective on the influences of hypoxic microenvironment on keratinocyte signaling pathways, which differ from its effects on signalling in other cells.

## Supporting Information

S1 Text Immunofluorescence staining. Cells cultured on collagen type IV-coated glass coverslips were fixed in 4% paraformaldehyde for 20 min. The fixed cells were subsequently incubated with the primary antibodies phospho-p70S6K (Thr389) and phospho-4E-BP1 (Thr70) (1:100; Cell Signalling, USA) overnight at 4°C. Then, the cells were washed with PBS and incubated with a secondary antibody conjugated to cyanine 3 (Cy3; 1:100; Beyotime, Shanghai, China) at 37°C for 1 h. The nuclei were stained with 4', 6-diamidino-2-phenylindole (DAPI; Hyclone, USA). The expression of phospho-p70S6K and phospho-4E-BP1 was observed under a Leica Confocal Microscope (Leica Microsystems, Wetzlar, Germany).
(DOCX)

S1 Fig Hypoxia increases the expression of mTORC1 downstream targets in the nucleus. (A) Immunofluorescence staining of anti-p70S6K phospho-Thr389 antibody (Cy3, red stain) and nuclear compartment (DAPI, blue stain) in normoxic and hypoxic HaCaT keratinocytes. Scale bar = 25 μm. (B) Immunofluorescence staining of anti-4E-BP1 phospho-Thr70 antibody (Cy3, red stain) and nuclear compartment (DAPI, blue stain) in normoxic and hypoxic HaCaT keratinocytes. Scale bar = 25 μm.
(TIF)

S2 Fig Transfection of shRNA against Raptor (shRaptor) effectively silences its expression. (A) MKs transfected with negative control (shNC) or shRNA against Raptor (shRaptor) were exposed to hypoxia for 6 hours and probed for Raptor. (B) Graph represents the means ± SD (n = 3) of the relative integrated signals. N, normoxia. *P< 0.05 versus the shNC group.
(TIF)

S1 Movie Inactivation of the mTORC1 pathway reduces hypoxia-induced keratinocyte motility and migration. Hypoxic cells were transfected with negative control (shNC) or shRNA against Raptor (shRaptor) or were incubated with or without Rapamycin. Time-lapse imaging was then performed for 3 hours. Scalebar = 50 μm. N, normoxia; H, hypoxia.
(AVI)

S2 Movie Activation of the AMPK pathway suppresses keratinocyte migration under hypoxia. Cells under normoxic or hypoxic culture conditions were treated with or without the AMPK activators Metformin (500 μM) and AICAR (0.5 mM). Sequential, time-lapse imaging was performed for 3 hours. Scalebar = 50 μm. N, normoxia; H, hypoxia.
(AVI)
