# Supplementary material for: Hypoxia Regulates mTORC1-Mediated Keratinocyte Motility and Migration via the AMPK Pathway
Source: PLoS One. 2017 Jan 9;12(1):e0169155. doi: 10.1371/journal.pone.0169155 (PMC5221764; doi:10.1371/journal.pone.0169155)
Supplement: S1 Text — Cells cultured on collagen type IV-coated glass coverslips were fixed in 4% paraformaldehyde for 20 min. The fixed cells were subsequently incubated with the primary antibodies phospho-p70S6K (Thr389) and phospho-4E-BP1 (Thr70) (1:100; Cell Signalling, USA) overnight at 4°C. Then, the cells were washed with PBS and incubated with a secondary antibody conjugated to cyanine 3 (Cy3; 1:100; Beyotime, Shanghai, China) at 37°C for 1 h. The nuclei were stained with 4', 6-diamidino-2-phenylindole (DAPI; Hyclone, USA). The expression of phospho-p70S6K and phospho-4E-BP1 was observed under a Leica Confocal Microscope (Leica Microsystems, Wetzlar, Germany). (DOCX) [file pone.0169155.s001.docx]

**Hypoxia Regulates mTORC1-mediated Keratinocyte Motility and Migration via the AMPK Pathway**

Tiantian Yan, Junhui Zhang, Di Tang, Xingyue Zhang, Xupin Jiang, Liping Zhao, Qiong Zhang, Dongxia Zhang, Yuesheng Huang**^*^**

Institute of Burn Research, State Key Laboratory of Trauma, Burns and Combined Injury, Southwest Hospital, Third Military Medical University, Chongqing, China.

*****Correspondence: Yuesheng Huang, E-mail: [yshuangtmmu@163.com](mailto:yshuangtmmu@163.com) (YH)

**Suppporting Information**

**Materials and Methods**

**Immunoﬂuorescence staining.** Cells cultured on collagen type IV-coated glass coverslips were ﬁxed in 4% paraformaldehyde for 20 min. The ﬁxed cells were subsequently incubated with the primary antibodies phospho-p70S6K (Thr389) and phospho-4E-BP1 (Thr70) (1:100; Cell Signalling, USA) overnight at 4°C. Then, the cells were washed with PBS and incubated with a secondary antibody conjugated to cyanine 3 (Cy3; 1:100; Beyotime, Shanghai, China) at 37°C for 1 h. The nuclei were stained with 4', 6-diamidino-2-phenylindole (DAPI; Hyclone, USA). The expression of phospho-p70S6K and phospho-4E-BP1 was observed under a Leica Confocal Microscope (Leica Microsystems, Wetzlar, Germany).
