# Supplementary figures and images for: Hypoxia Regulates mTORC1-Mediated Keratinocyte Motility and Migration via the AMPK Pathway
Source: PLoS One. 2017 Jan 9;12(1):e0169155. doi: 10.1371/journal.pone.0169155 (PMC5221764; doi:10.1371/journal.pone.0169155)

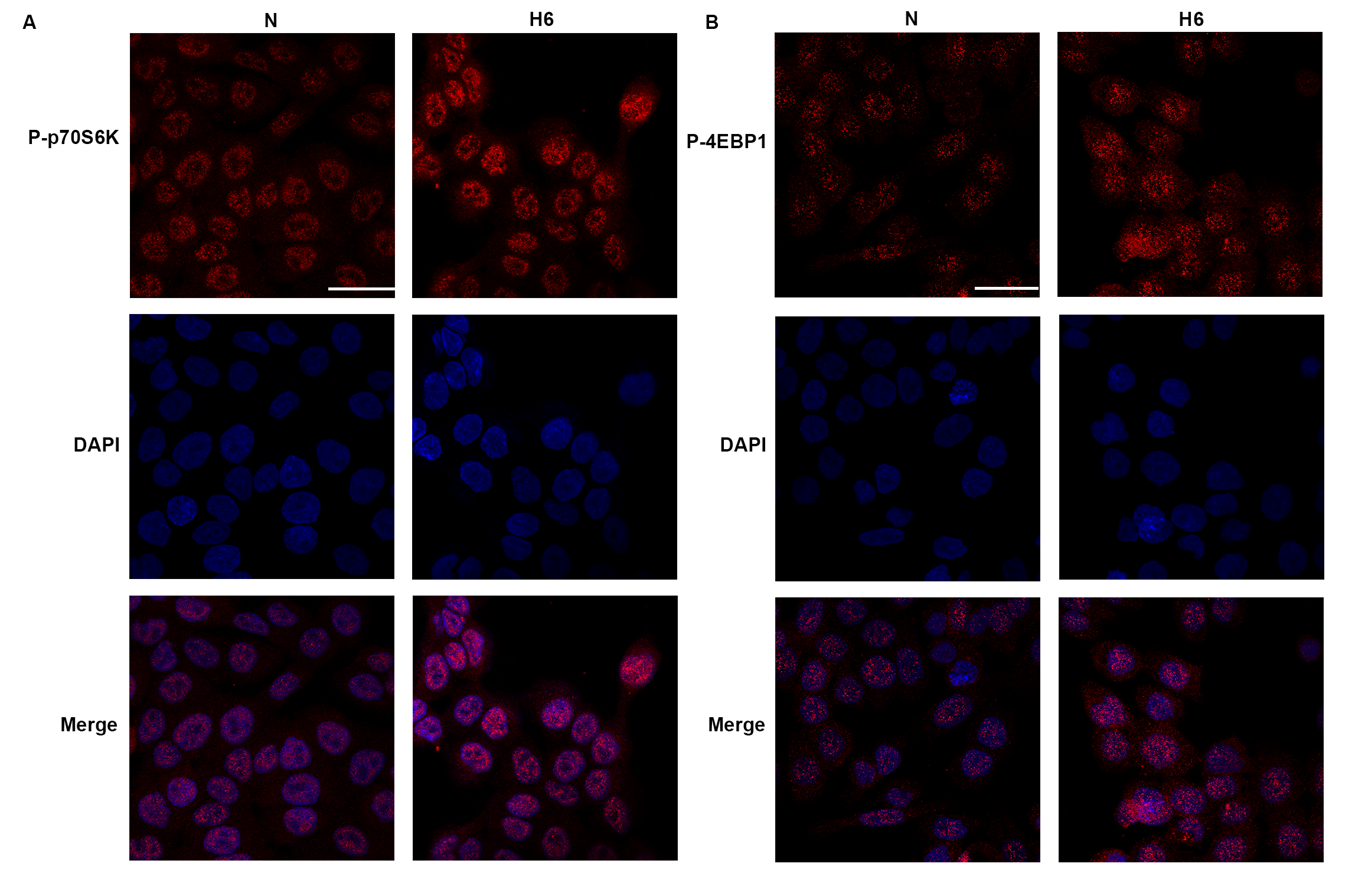

Supplement: S1 Fig — (A) Immunofluorescence staining of anti-p70S6K phospho-Thr389 antibody (Cy3, red stain) and nuclear compartment (DAPI, blue stain) in normoxic and hypoxic HaCaT keratinocytes. Scale bar = 25 μm. (B) Immunofluorescence staining of anti-4E-BP1 phospho-Thr70 antibody (Cy3, red stain) and nuclear compartment (DAPI, blue stain) in normoxic and hypoxic HaCaT keratinocytes. Scale bar = 25 μm. (TIF) [file pone.0169155.s002.tif]

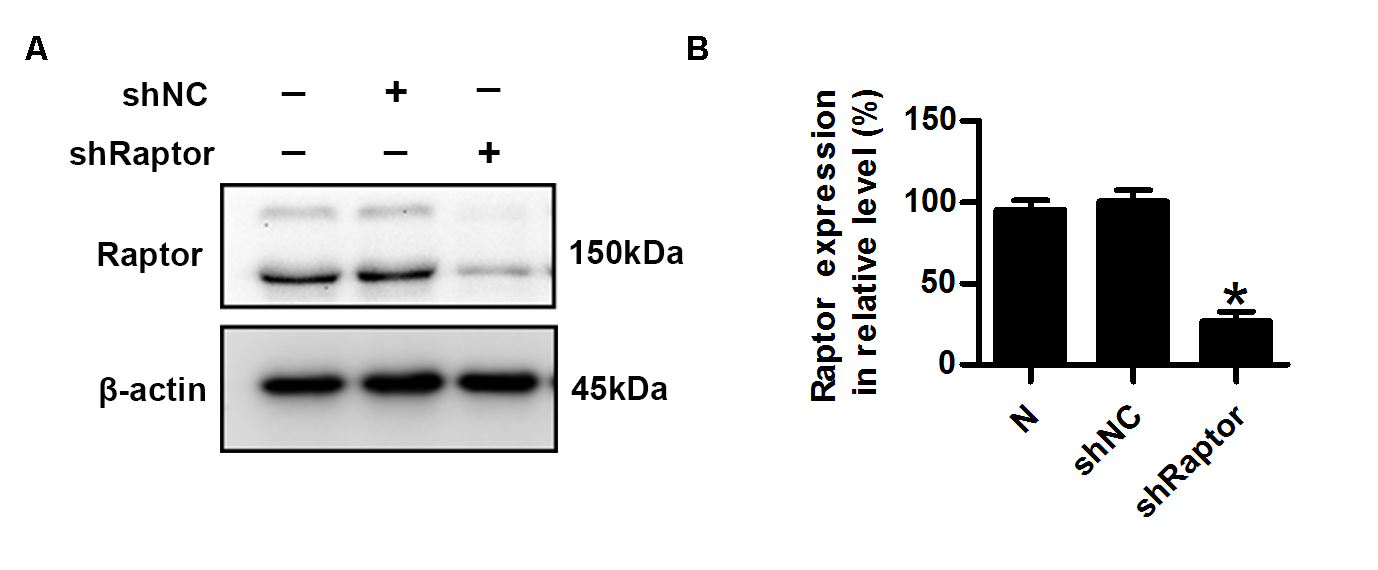

Supplement: S2 Fig — (A) MKs transfected with negative control (shNC) or shRNA against Raptor (shRaptor) were exposed to hypoxia for 6 hours and probed for Raptor. (B) Graph represents the means ± SD (n = 3) of the relative integrated signals. N, normoxia. *P< 0.05 versus the shNC group. (TIF) [file pone.0169155.s003.tif]
